# Bis(acetyl­acetonato-κ^2^ *O*,*O*′)[copper(II)nickel(II)(0.31/0.69)]: a mixed-metal complex

**DOI:** 10.1107/S160053681002756X

**Published:** 2010-07-17

**Authors:** Muhammad Shahid, Mazhar Hamid, Muhammad Mazhar, Mohammad Azad Malik, James Raftery

**Affiliations:** a Department of Chemistry, Quaid-i-Azam University, Islamabad 45320, Pakistan; b Department of Chemistry, University of Malaya, 50603 Kuala Lumpur, Malaysia; c The School of Chemistry, The University of Manchester, Oxford Road, Manchester M13 9PL, England

## Abstract

The title complex, [Cu_0.31_Ni_0.69_(C_5_H_7_O_2_)_2_], was isolated from the reaction of bis­(*N*,*N*-dimethyamino­ethanol)copper(II) with bis­(acetyl­acetonato)nickel(II), which yielded crystals with mixed sites at the central metal position; the refined copper–nickel occupancy ratio is 0.31 (4):0.69 (4). Two acetyl­acetonate ligands, related by a centre of symmetry, are coordinated to the central metal atom in a square-planar configuration while the methyne C atoms of the acetyl­acetonate ligands, *ca* 3.02 Å away, are orthogonal to this plane at the metal site.

## Related literature

For heterobimetallic complexes of copper and nickel, see: Hamid *et al.* (2006 ▶). For disorder in metal sites, see: Werndrup & Kessler (2001 ▶). For applications of mixed-metal ceramic oxides, see: Auciello & Ramesh(1996 ▶) and references therein. For mixed copper/nickel oxide catalysts, see: Kessler *et al.* (2001 ▶). For the synthesis of Cu(dmae)_2_ (dmae = *N*,*N*-dimethyl­aminoethanolato), see: Johnson *et al.* (2001 ▶). For the crystal structure of Cu(acac)_2 _(acac = acetyl­acetonato), see: LeBrun *et al.* (1986 ▶). For the crystal structure of Ni(acac)_2_·2H_2_O, see: Zhou *et al.* (2001 ▶). For the O—Cu/Ni—O chelate bite angle in related complexes, see: Aruffo *et al.* (1983 ▶).
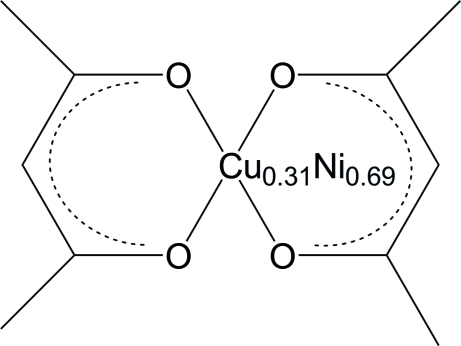


## Experimental

### 

#### Crystal data

[Cu_0.31_Ni_0.69_(C_5_H_7_O_2_)_2_]
*M* *_r_* = 258.40
Monoclinic, 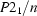

*a* = 10.265 (1) Å
*b* = 4.6300 (5) Å
*c* = 11.2830 (11) Å
β = 92.431 (2)°
*V* = 535.76 (9) Å^3^
*Z* = 2
Mo *K*α radiation
μ = 1.87 mm^−1^
*T* = 100 K
0.45 × 0.45 × 0.20 mm

#### Data collection

Bruker SMART CCD area-detector diffractometer
Absorption correction: multi-scan (*SADABS*; Sheldrick, 2008 ▶) *T*_min_ = 0.487, *T*_max_ = 0.706
3086 measured reflections
1242 independent reflections
1159 reflections with *I* > 2σ(*I*)
*R*_int_ = 0.013

#### Refinement

*R*[*F* ^2^ > 2σ(*F* ^2^)] = 0.022
*wR*(*F* ^2^) = 0.059
*S* = 1.07
1242 reflections
73 parameters
H-atom parameters constrained
Δρ_max_ = 0.37 e Å^−3^
Δρ_min_ = −0.35 e Å^−3^

### 

Data collection: *SMART* (Bruker, 2007 ▶); cell refinement: *SAINT* (Bruker, 2007 ▶); data reduction: *SAINT*; program(s) used to solve structure: *SIR2004* (Burla *et al.*, 2005 ▶); program(s) used to refine structure: *SHELXL97* (Sheldrick, 2008 ▶); molecular graphics: *SHELXTL* (Sheldrick, 2008 ▶); software used to prepare material for publication: *SHELXTL*.

## Supplementary Material

Crystal structure: contains datablocks I, global. DOI: 10.1107/S160053681002756X/su2192sup1.cif

Structure factors: contains datablocks I. DOI: 10.1107/S160053681002756X/su2192Isup2.hkl

Additional supplementary materials:  crystallographic information; 3D view; checkCIF report

## Figures and Tables

**Fig. 1.**
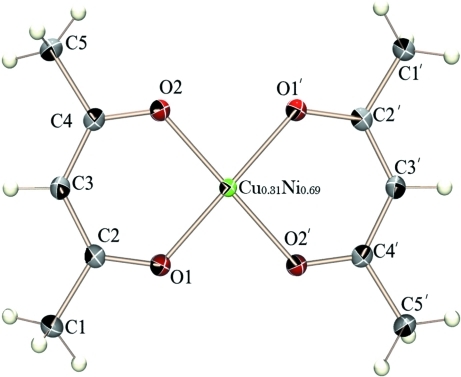
View of the molecular structure of the title molecule with displacement ellipsoids drawn at the 50% probability level.

## supplementary crystallographic information

### Comment

Heterobimetallic complexes of copper and nickel have already been reported as precursors for chemical vapor deposition of ceramic material thin films (Hamid *et al.*, 2006). Mixed metal ceramic oxides have multiple compositions and crystal structures, which results in a diversity of properties leading to a vast variety of potential applications (Auciello *et al.*, 1996, and references therein). For example, a mixed copper/nickel oxide catalyst  was deposited on a zeolite support and was shown to have extremely high activity towards methanol oxidation (Kessler *et al.*, 2001).

The title complex was synthesized by the reaction of Cu (dmae)_2_ (dmae = N,N-dimethylaminoethanolato) (Johnson *et al.*, 2001) with Ni(acac)_2_.2H_2_O (acac = acetylacetonato) in toluene. In contrast to the formation of the oligomeric bimetallic complex (Hamid *et al.*, 2006), the title compound crystallized out with the central position partially occupied by Cu and partially by Ni, with a refined Cu:Ni occupancy ratio of 0.31 (4):0.69 (4). A similar type of disorder in the metal site was observed previously in Ni(Ni_0.25_Cu_0.75_)_2_(µ_3_-OH)(µ-OAc)_2_(η^1^-OAc)_2_(µ,η^2^–ORN)_2_ (η^2^-R^N^OH)][R^N^–OH=(CH_3_)_2_N(CH_2_)(CHOH)CH_3_)] (Werndrup *et al.*, 2001), where the two metal sites were occupied by 75% Cu and 25% Ni. The distribution of two metals at the central position in the title complex is random which means some of the molecules would have each of the two Cu and Ni atoms, or in other words if we consider it to be systematic, in every molecule the position will be occupied by exactly 0.31 Cu and 0.69 Ni atoms.

The molecular structure of the title complex is shown in Figure 1. The geometry of the title complex is square planer, similar to that of Cu(acac)_2_ (LeBrun *et al.*, 1986) where two ligands coordinate to the metal atom in the same plane, while in the nickel(II) complex, Ni(acac)_2_ (Zhou *et al.*, 2001), which crystallized with two coordinated water molecules, the metal has an octahedral coordination sphere. The metal to oxygen (O1, O2) bond distances [1.9196 (10) and 1.9225 (10) Å] are slightly longer than those in Cu(acac)_2_ [1.914 (4), 1.914 (4)Å] but shorter than the average value found in Ni(acac)_2_ [2.0147Å]. The O—Cu/Ni—O chelate bite angle is 93.72 (4)° which is comparable to that found in Cu(acac)_2_ [93.2 (2)°] and other complexes of this type (Aruffo *et al.*, 1983). The chelate bite angles are of course larger than those in the octahedral nickel(II) complex mentioned above [91.65 (8)°, 89.99 (8)°].

### Experimental

Bis(*N*,*N*-dimethylaminoethanolatoκ^2^ O, N) copper(II) (0.5 g, 2.1 mmol) and bis(acetylacetonato κ^2^ O, O') nickel(II) (0.54 g, 2.1 mmol) were reacted in 20 ml toluene as a solvent. After stirring for two hours, the solution was cannula filtered to remove unreacted reagents. Slow evaporation of the filtrate gave block-like blue crystals, suitable for single-crystal X-ray analysis, after two weeks.

### Refinement

The H-atoms were included in calculated positions, with C—H = 0.95(CH), 0.99(CH_2_) & 0.98(CH_3_) Å, with *U*_iso_(H) = k × *U*_eq_(C), where k = 1.5 for CH_3_ H-atoms and 1.2 for all other H-atoms.

The occupancy of the metal site was examined under three assumptions: All Ni gave R1(>4sig)= 0.0227, R1(all)= 0.0243; All Cu gave R1(>4sig)= 0.0236, R1(all)= 0.0252. Variable Ni:Cu ratio [which converged to 69 (4):31 (4)] gave R1(>4sig)= 0.0221, R1(all)= 0.0238. Elemental analysis of the Cu/Ni with a ICP-OES Fisons Horizon Spectrometer has: ratio Cu:Ni (31:69); Cu calulated 7.62%: found 7.86%; Ni calculated 15.68%: found 15.23%. The agreement is surprisingly good considering that Cu and Ni differ by only one electron.

### Crystal data

**Table:**

[Cu_0.31_Ni_0.69_(C_5_H_7_O_2_)_2_]	*F*(000) = 269
*M**_r_* = 258.40	*D*_x_ = 1.602 Mg m^−^^3^
Monoclinic, *P*2_1_/*n*	Mo *K*α radiation, λ = 0.71073 Å
Hall symbol: -P 2yn	Cell parameters from 2222 reflections
*a* = 10.265 (1) Å	θ = 2.6–28.2°
*b* = 4.6300 (5) Å	µ = 1.87 mm^−^^1^
*c* = 11.2830 (11) Å	*T* = 100 K
β = 92.431 (2)°	Block, blue
*V* = 535.76 (9) Å^3^	0.45 × 0.45 × 0.20 mm
*Z* = 2	

### Data collection

**Table:**

Bruker SMART CCD area-detector diffractometer	1242 independent reflections
Radiation source: fine-focus sealed tube	1159 reflections with *I* > 2σ(*I*)
graphite	*R*_int_ = 0.013
phi and ω scans	θ_max_ = 28.2°, θ_min_ = 2.6°
Absorption correction: multi-scan (*SADABS*; Sheldrick, 2008)	*h* = −9→13
*T*_min_ = 0.487, *T*_max_ = 0.706	*k* = −6→5
3086 measured reflections	*l* = −13→14

### Refinement

**Table:**

Refinement on *F*^2^	Primary atom site location: structure-invariant direct methods
Least-squares matrix: full	Secondary atom site location: difference Fourier map
*R*[*F*^2^ > 2σ(*F*^2^)] = 0.022	Hydrogen site location: inferred from neighbouring sites
*wR*(*F*^2^) = 0.059	H-atom parameters constrained
*S* = 1.07	*w* = 1/[σ^2^(*F*_o_^2^) + (0.0305*P*)^2^ + 0.3448*P*] where *P* = (*F*_o_^2^ + 2*F*_c_^2^)/3
1242 reflections	(Δ/σ)_max_ < 0.001
73 parameters	Δρ_max_ = 0.37 e Å^−^^3^
0 restraints	Δρ_min_ = −0.35 e Å^−^^3^

### Special details

**Table:**

Geometry. All e.s.d.'s (except the e.s.d. in the dihedral angle between two l.s. planes) are estimated using the full covariance matrix. The cell e.s.d.'s are taken into account individually in the estimation of e.s.d.'s in distances, angles and torsion angles; correlations between e.s.d.'s in cell parameters are only used when they are defined by crystal symmetry. An approximate (isotropic) treatment of cell e.s.d.'s is used for estimating e.s.d.'s involving l.s. planes.
Refinement. Refinement of *F*^2^ against ALL reflections. The weighted *R*-factor *wR* and goodness of fit *S* are based on *F*^2^, conventional *R*-factors *R* are based on *F*, with *F* set to zero for negative *F*^2^. The threshold expression of *F*^2^ > σ(*F*^2^) is used only for calculating *R*-factors(gt) *etc*. and is not relevant to the choice of reflections for refinement. *R*-factors based on *F*^2^ are statistically about twice as large as those based on *F*, and *R*- factors based on ALL data will be even larger.

### Fractional atomic coordinates and isotropic or equivalent isotropic displacement parameters (Å^2^)

**Table:**

	*x*	*y*	*z*	*U*_iso_*/*U*_eq_	Occ. (<1)
C1	0.81881 (15)	0.5644 (4)	0.55275 (14)	0.0207 (3)	
H1A	0.8798	0.4228	0.5220	0.031*	
H1B	0.8547	0.6431	0.6279	0.031*	
H1C	0.8056	0.7213	0.4952	0.031*	
C2	0.69044 (14)	0.4199 (3)	0.57303 (13)	0.0167 (3)	
C3	0.61101 (16)	0.5306 (3)	0.65990 (14)	0.0184 (3)	
H3	0.6416	0.6941	0.7037	0.022*	
C4	0.48909 (14)	0.4159 (3)	0.68655 (13)	0.0170 (3)	
C5	0.41401 (16)	0.5520 (4)	0.78376 (14)	0.0219 (3)	
H5A	0.3603	0.7100	0.7508	0.033*	
H5B	0.4751	0.6279	0.8452	0.033*	
H5C	0.3578	0.4065	0.8186	0.033*	
Ni1	0.5000	0.0000	0.5000	0.01368 (10)	0.69 (4)
Cu1	0.5000	0.0000	0.5000	0.01368 (10)	0.31 (4)
O1	0.66241 (10)	0.2061 (2)	0.50564 (9)	0.0185 (2)	
O2	0.43456 (10)	0.2013 (2)	0.63411 (9)	0.0182 (2)	

### Atomic displacement parameters (Å^2^)

**Table:**

	*U*^11^	*U*^22^	*U*^33^	*U*^12^	*U*^13^	*U*^23^
C1	0.0171 (7)	0.0222 (7)	0.0229 (7)	−0.0021 (6)	0.0022 (6)	−0.0017 (6)
C2	0.0168 (7)	0.0163 (7)	0.0170 (7)	0.0004 (6)	−0.0016 (5)	0.0029 (5)
C3	0.0196 (7)	0.0186 (7)	0.0172 (7)	−0.0019 (6)	0.0008 (5)	−0.0023 (5)
C4	0.0192 (7)	0.0167 (7)	0.0151 (7)	0.0016 (6)	0.0008 (5)	0.0013 (5)
C5	0.0228 (8)	0.0230 (8)	0.0204 (7)	−0.0013 (6)	0.0058 (6)	−0.0039 (6)
Ni1	0.01363 (14)	0.01262 (15)	0.01500 (15)	−0.00132 (9)	0.00309 (9)	−0.00194 (9)
Cu1	0.01363 (14)	0.01262 (15)	0.01500 (15)	−0.00132 (9)	0.00309 (9)	−0.00194 (9)
O1	0.0179 (5)	0.0171 (5)	0.0207 (5)	−0.0008 (4)	0.0029 (4)	−0.0019 (4)
O2	0.0188 (5)	0.0165 (5)	0.0195 (5)	−0.0015 (4)	0.0037 (4)	−0.0015 (4)

### Geometric parameters (Å, °)

**Table:**

C1—C2	1.504 (2)	C4—C5	1.505 (2)
C1—H1A	0.9800	C5—H5A	0.9800
C1—H1B	0.9800	C5—H5B	0.9800
C1—H1C	0.9800	C5—H5C	0.9800
C2—O1	1.2737 (18)	Ni1—O1^i^	1.9196 (10)
C2—C3	1.399 (2)	Ni1—O1	1.9196 (10)
C3—C4	1.404 (2)	Ni1—O2^i^	1.9225 (10)
C3—H3	0.9500	Ni1—O2	1.9226 (10)
C4—O2	1.2737 (18)		
			
C2—C1—H1A	109.5	C4—C5—H5A	109.5
C2—C1—H1B	109.5	C4—C5—H5B	109.5
H1A—C1—H1B	109.5	H5A—C5—H5B	109.5
C2—C1—H1C	109.5	C4—C5—H5C	109.5
H1A—C1—H1C	109.5	H5A—C5—H5C	109.5
H1B—C1—H1C	109.5	H5B—C5—H5C	109.5
O1—C2—C3	125.43 (14)	O1^i^—Ni1—O1	179.999 (1)
O1—C2—C1	115.60 (13)	O1^i^—Ni1—O2^i^	93.72 (4)
C3—C2—C1	118.95 (14)	O1—Ni1—O2^i^	86.28 (4)
C2—C3—C4	124.25 (14)	O1^i^—Ni1—O2	86.28 (4)
C2—C3—H3	117.9	O1—Ni1—O2	93.72 (4)
C4—C3—H3	117.9	O2^i^—Ni1—O2	180.0
O2—C4—C3	125.02 (14)	C2—O1—Ni1	125.45 (10)
O2—C4—C5	115.87 (13)	C4—O2—Ni1	125.62 (9)
C3—C4—C5	119.10 (14)		
			
O1—C2—C3—C4	1.3 (3)	O2^i^—Ni1—O1—C2	172.90 (12)
C1—C2—C3—C4	179.64 (14)	O2—Ni1—O1—C2	−7.10 (12)
C2—C3—C4—O2	−1.3 (2)	C3—C4—O2—Ni1	−4.3 (2)
C2—C3—C4—C5	178.91 (15)	C5—C4—O2—Ni1	175.47 (10)
C3—C2—O1—Ni1	4.2 (2)	O1^i^—Ni1—O2—C4	−172.85 (12)
C1—C2—O1—Ni1	−174.11 (10)	O1—Ni1—O2—C4	7.15 (12)
O1^i^—Ni1—O1—C2	170 (6)	O2^i^—Ni1—O2—C4	98 (29)

Symmetry codes: (i) −*x*+1, −*y*, −*z*+1.
